# Impact of human papillomaviruses (HPV) on recurrence rate and malignant progression of sinonasal papillomas

**DOI:** 10.1002/cam4.3642

**Published:** 2020-12-22

**Authors:** Anja Paehler vor der Holte, Inger Fangk, Sabine Glombitza, Ludwig Wilkens, Hans J. Welkoborsky

**Affiliations:** ^1^ Department of Otorhinolaryngology, Head and Neck Surgery Nordstadt Clinic Academic Hospital Hanover Germany; ^2^ Department of Pathology and Molecular Pathology Nordstadt Clinic Academic Hospital Hanover Germany

**Keywords:** human papillomavirus, inverted papilloma, Paranasal sinus neoplasms, risk factors

## Abstract

Sinonasal papillomas are characterized by their potential for frequent recurrences and malignant progression. Currently, the role of human papillomavirus (HPV) infection in sinonasal papillomas is unclear. A study was conducted to elucidate the impact of HPV infection on recurrence and malignant progression of sinonasal papillomas. One hundred and seven patients with 151 tumors could be examined. One hundred and one patients suffered from benign papilloma, mostly inverted papillomas (IP); six patients suffered from carcinomas in situ and squamous cell carcinomas (SCC) ex‐IP. Recurrent IP were more often HPV‐positive than non‐recurrent tumors (38.8% vs. 60%–65%). Low‐risk (LR) HPV infection (especially HPV 6) increased the risk of tumor recurrences (*p* = 0.0385 and *p* = 0.0556, respectively). IP and oncocytic papillomas (both lesions are known for their malignant potential) were more often high‐risk (HR) HPV‐positive (15.5% and 16.7%) than fungiform papilloma (which usually does not progress to carcinoma). CIS and SCC ex‐IP displayed higher HPV rates than benign IP (83.3% vs. 38.8%), especially higher rates of HR‐HPV (66.7% vs. 23.8%, *p* = 0.0415). Data from this study endorse the hypothesis that recurrence of sinonasal papillomas is promoted by LR‐HPV infection and that malignant progression of IP is promoted by HR‐HPV infection.

## Graphical abstract

The impact of HPV infection on recurrences and malignant transformation of sinonasal papillomas is elucidated. HPV infection rate is significantly higher in recurrent papillomas than in non‐recurrent and the highest in papillomas with malignant progression. Most frequent HPV subtypes are HPV 6 in benign recurrent tumors, and HPV 16, 45, and 66 in malignant tumors, along with HPV 90 which is currently described for the first time in sinonasal papillomas. The data suggest that HPV infection is a risk factor for tumor recurrences. Malignant progression seems to be supported by high‐risk HPV genotypes (i.e., 16, 45, 66, and probably 90).

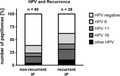

**Statements**

1. The authors do not have any conflict of interest and therefore nothing to disclose.
2. This is a completely anonymized cohort study. All patients with clinical or histological/molecular biological data included in this study had to undergo the procedure regardless the scientific evaluation of the histological and molecular biological findings and gave their informed consent prior to the examinations and publication of results.
3. All procedures performed in this examination involving human participants were in accordance with the ethical standards and with the Declaration of Helsinki and its later amendments.

## 1 INTRODUCTION

Papillomas in the sinonasal tract are usually arising from the Schneiderian membrane and are therefore called “Schneiderian papillomas”. According to their histopathological characteristics, three different papilloma groups can be distinguished: (a) inverted papilloma (IP), which is the most frequent group preferentially affecting patients between 40 through 70 years of age, (b) fungiform (exophytic) papilloma (FP), which affects patients younger in average (20 through 50 years of age), and (c) oncocytic papilloma (OCP), which is very rare and usually affects patients older than 50 years.^1^ IP and FP are more often observed in male patients, rather than OCP, which occurs equally among male and female patients.^1^ From a clinical point of view, papillomas demonstrate three main characteristics: (a) a destructive growth pattern into the local tissue and even into bony structures, (b) frequent development of tumor recurrences, and (c) transformation into malignant conditions (particularly IP and OCP).^1^ Recurrent papillomas usually develop within 2 to 3 years after initial treatment. However, recurrences after a long disease‐free period can also be observed during long‐term follow‐up.^2^ As far as malignant conditions are concerned, IP and OCP are usually associated with squamous cell carcinoma rather than to other malignant tumors.^3^ FP usually does not progress to malignancy.^1^

Human papillomaviruses (HPV) are DNA viruses infecting mucous membrane and skin cells. Oncogenically, they express a variety of viral antigens or oncoproteins that can lead to uncontrolled cell proliferation in infected tissue.^4^ The infection by certain HPV genotypes may lead to malignant progression due to uncontrolled expression of their particular viral oncogenes.^5^ Low‐risk HPV types (LR‐HPV) usually do not lead to malignancy (e.g., HPV types 6 or 11), while the likelihood of carcinogenesis in high‐risk HPV types (HR‐HPV) (e.g., HPV 16 or 18) is significantly increased (Table 1).^6^, ^7^, ^8^ HPV infections are responsible for or associated with a wealth of benign and malignant tumorous lesions in the head and neck area, that is, skin warts, laryngeal papillomas, or squamous cell carcinoma of the oropharynx.^9^, ^10^, ^11^ Currently, the possible impact of HPV in the development and progression of sinonasal papillomas is controversially discussed in recent literature.^12^, ^13^, ^14^, ^15^, ^16^, ^17^, ^18^, ^19^

**TABLE 1:** Classification of Human Papillomaviruses (HPV)

Classification of Human Papillomaviruses	
Low risk (LR)	6, 11, 40, 42, 43, 44, 54, 61, 70, 72, 81, CP6108
Probable low risk (pLR)	32, 62, 74, 83, 84, 86, 87, 91, CP8304
Unknown / undetermined risk (UR)	71, 90, IA09, JC9710, W13B
Probable high risk (pHR)	26, 30, 34, 53, 66, 67, 69, MM4
High risk (HR)	16, 18, 31, 33, 35, 39, 45, 51, 52, 56, 58, 59, 66, 68, 73, 82

HPV genotypes are classified according to their cancerogenic potential in lesions of the uterine cervix: there are low risk types, probable low risk, unknown risk, probable high risk, and high risk types.^5^, ^6^, ^7^

The present paper describes several HPV genotypes identified in sinonasal papillomas and discusses whether such an infection might be regarded as a risk factor for tumor recurrences and malignant progression.

## 2 METHODS

### 2.1 Monocentric retrospective study

For this retrospective study, the operative specimens of 107 patients sinonasal papilloma were examined, who were admitted to the Department of Otorhinolaryngology, Head and Neck Surgery of a tertiary referral teaching hospital. The specimens were collected in a 10‐year period (January 2000 through December 2010). If patients presented with recurrent papillomas within this time (even if initial diagnosis was prior to January 2000), or if patients suffered from recurrence after 2010, samples of their tumors were also studied. In the end, the earliest tumor dated back to 1996, the latest tumor recurrence was treated in May 2017. The clinical follow‐up was 3.1 years in the average.

To conduct the retrospective analysis, patients were identified by the hospital information system searching for ICD code D14.0. Finally, 107 patients with histologically confirmed sinonasal papillomas or carcinoma ex‐papilloma could be included in this study.

Some patients underwent functional endonasal sinus surgery for non‐suspicious chronic rhino‐sinusitis with nasal or sinus polyps. The diagnosis of a papilloma was then made histopathologically by investigating the specimens. In these cases, a control CT scan of the paranasal sinuses was performed about 3 months following initial surgery, assuming that wound healing is completed by then. If suspicious masses were found in this CT scan or endoscopically during patient examination, patients underwent revision surgery under oncologic conditions. For this study, these cases were counted as persisting papillomas, not as real tumor recurrences.

The specimens were obtained during surgery, were fixed in formalin, and then embedded in paraffin. 5‐µm slices were cut from the paraffin‐embedded tissues, dewaxed, and stained with hematoxylin and eosin (HE) for histopathological examination. According to histological results, patients were divided into four groups: (a) patients with inverted papilloma (IP), (b) fungiform papilloma (FP), (c) oncocytic papilloma (OCP), and (d) carcinoma in situ ex‐papilloma (CIS ex‐IP) or squamous cell carcinoma (SCC ex‐IP).

This patient cohort was examined extensively for their clinical risk factors of papilloma recurrence previously.^2^ However, the present study focusses mainly on the potential impact of HPV infection on tumor recurrence and malignant progression.

### 2.2 DNA extraction and check for DNA quality

Tumor tissue was identified microscopically in HE‐stained specimens and harvested. DNA was extracted using the spin‐column‐based QiAamp^®^ DNA FFPE Tissue Kit (Qiagen Cie, Hilden, Germany) as reported in detail previously.^2^ For measuring the DNA concentration, the NanoDrop ND‐1000 spectrophotometer (Peqlab, Erlangen) was used. DNA purity was then confirmed by 230/260 and 260/280 ratio. To assess the DNA quality, GAPDH amplification in a multiplex PCR (Table 2) followed by gel electrophoresis (4% agarose gel) was performed.

**TABLE 2:** Information on primers for PCR

	Sequence (5′→3′)	Concentration	Transcript length	Comment
GAPDH−105	for: GGCTGAGAACGGGAAGCTTG rev: ATCCTAGTTGCCTCCCCAAA	0.24 µM	105 bp	
GAPDH−236	for: CGGGTCTTTGCAGTCGTATG rev: GCGAAAGGAAAGAAAGCGTC	0.6 µM	236 bp	
GAPDH−299	for: AGGTGAGACATTCTTGCTGG rev: TCCACTAACCAGTCAGCGTC	1.2 µM	299 bp	
GAPDH−411	for: TGAATGGGCAGCCGTTAGGAAAGC rev: AGACACCCAATCCTCCCGGTGACA	0.3 µM	411 bp	
MY09 MY11	for: CGTCCMARRGGAWACTGATC rev: GCMCAGGGWCATAAYAATGG	0.2 µM	ca. 450 bp	M = A + C; R = A + G; W = A + T; Y = C + T
125	—	0.4 µM	125–155 bp	Primers were provided by the HPV Type 3.5 LCD array kit (Chipron GmbH).

The table displays the primer sequences, their concentration in the PCR reaction, and length of the transcript.

### 2.3 PCR

The PCR procedure for detection of HPV DNA was performed as reported previously.^2^ In brief, 10–20 ng DNA were used as template. Each reaction additionally contained PCR buffer, dNTP mix (Biozym Scientific, Hessisch Oldendorf, Germany), HotStar Taq^®^ polymerase (Qiagen, Hilden), and an appropriate amount of primers (Table 2). Homogeneity and identity of PCR products were verified by agarose gel electrophoresis.

### 2.4 DNA microarrays

The HPV Type 3.5 LCD‐array kit (Chipron GmbH) was used for HPV genotyping as reported previously.^2^ In brief, HPV genes were amplified by L1 consensus primer pairs. Amplicons were distributed onto the LCD‐chip. After reverse hybridization to genotype‐specific capture probes, biotinylated primers were labeled by streptavidin and horseradish peroxidase. Substrate oxidation formed blue precipitates and the subsequent color change was read on a spectrophotometer. HPV types were analyzed using the Slide Reader Software from Chipron GmbH (Berlin).

### 2.5 Statistics

All descriptive statistics were performed using the GraphPad Prism 5 software. To analyze the impact of the different HPV genotypes on recurrence of sinonasal papillomas, data were compared by univariate logistic regression using the R project for statistical computing. Fisher's exact test was used when investigating the risk of HPV infection and malignant progression of inverted papillomas (GraphPad Prism 5 software). Statistical significance was assumed at p‐values of less than 0.05.

## 3 RESULTS

### 3.1 Sinonasal papillomas recur frequently. Inverted papillomas display the tendency for malignant progression. Benign papillomas and carcinomas ex‐IP favor the male gender.

One hundred and seven patients with 166 tumors were included in this study. Six of the 107 patients (5.3%) displayed 12 tumors with malignant criteria (7.2%), such as carcinoma in situ (CIS) and squamous cell carcinoma (SCC), 101 patients suffered from benign papillomas. In regard to benign papillomas, most of the patients presented with inverted papilloma (IP) (91 patients = 85%), followed by fungiform papilloma (FP) (7 patients = 6.5%), and oncocytic papilloma (OCP) (2 patients = 1.9%).^2^ For one patient, it was not clear whether she suffered from IP or FP, so she was not analyzed as part of these groups.^2^ Schneiderian papillomas favored the male gender (men: women ratio = 3.5), which was also observed for carcinomas ex‐papillomas (men: women ratio = 2). The difference in sex ratios between benign and malignant tumors was statistically not significant (*p* = 0.6161).

All malignant tumors arose from inverted papillomas. Median age of patients with benign IP at the time of initial diagnosis was 59 years (range 26 through 83 years).^2^ Carcinoma ex‐IP patients displayed a median age of 55 years (range 53 through 85 years), when they were first diagnosed.

Most papillomas recurred within the first 5 years (roughly 74%); however, there were also late recurrences/secondary papillomas after more than 15 years.

### 3.2 Recurrent IP were more often hpv‐positive than non‐recurrent papillomas. lr‐hpv (especially hpv 6) infections increased the risk to suffer from recurrent schneiderian papillomas.

**FIGURE 1:**
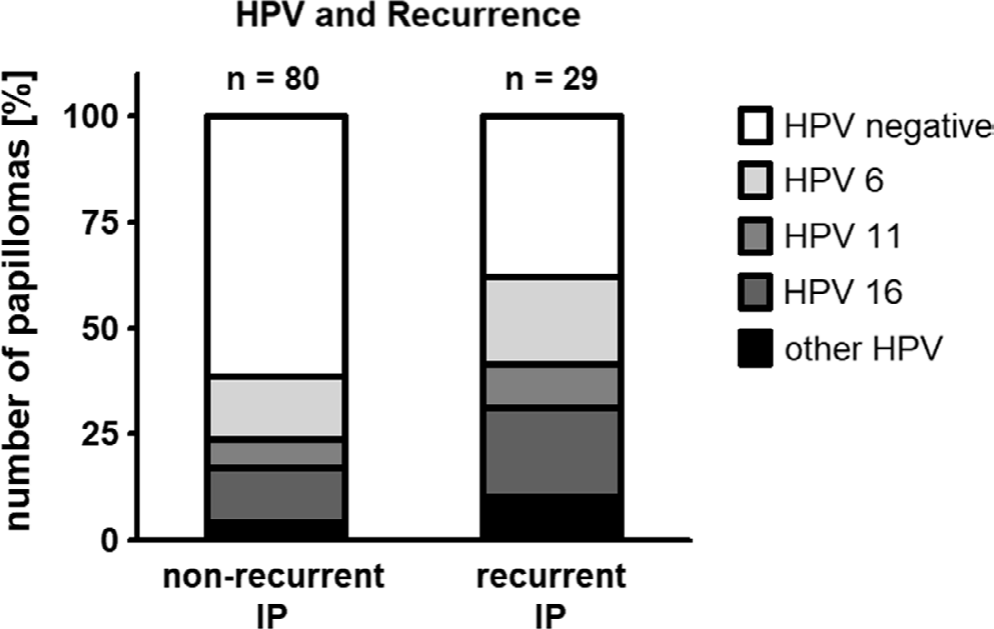
Recurrent inverted papillomas (IP) displayed a higher human papilloma virus (HPV) infection rate than non‐recurrent IP. This graph shows the number of HPV‐positive (grey to black bars) or HPV‐negative (white bars) IP depending on tumor recurrence. For purposes of clarity, HPV rates were weighted in this graph to account for co‐infections with multiple HPV types. The overall HPV rate in recurrent IP was significantly higher (38.8% vs. 62.1%, *p* = 0.0504). Especially the rate of HPV 6, 11, 16, 42, and 58 increased in recurrent papillomas from 1.4‐ to 5.9‐fold. Logistic regression analysis revealed that low‐risk HPV infections are a risk factor for recurrence of sinonasal papillomas (*p* = 0.0385). However, none of the above mentioned genotypes individually was a significant risk factor for recurrence of IP (*p* = 0.0556 up to 0.8128)

Twenty‐nine of the 91 patients with inverted papilloma suffered from at least one tumor recurrence (31.9%). Recurrence rates for men and women did not differ significantly (*p* = 0.4362). Overall, IP reappeared quite often: 20 patients suffered from one tumor recurrence (22%), five patients from two (5.5%), three patients from three (3.3%), and one patient from more than three tumor recurrences (1.1%). Not all specimens could be investigated for HPV infection, because sometimes patients were referred to our hospital only after tumors recurred and tissue samples from the initial surgery could not be gathered. A total of 80 initially diagnosed IP, 21 first recurrent tumors, and five second recurrent tumors could be examined for HPV infection. Recurrent tumors were more often HPV‐positive than non‐recurrent IP (*p* = 0.0504): at initial diagnosis, 38.8% of inverted papillomas were HPV‐positive (31 of 80 IP); 65% of first recurrent tumors were HPV‐positive (13 of 21 IP), and 60% of second recurrent tumors (3 of 5 IP). Especially, the rate of HPV 11, 16, 42, and 58 increased in recurrent papillomas; however, none of these HPV types individually revealed a statistically significant higher detection rate in recurrent IP (*p* = 0.3079 up to 0.8128) (Figure 1). However, regression analysis revealed, that the sum of LR‐HPV infections (especially HPV 6) increased the risk to suffer from recurrent papillomas (*p* = 0.0385 for LR‐HPV and *p* = 0.0556 for HPV 6) (Figure 1). Co‐infections with multiple HPV types did not increase recurrence rates compared to single HPV infections (*p* = 0.1483).

Two of the seven patients with fungiform papilloma suffered from tumor recurrence (28.6%). Both patients were male. One of them presented with one recurrent FP, while FP reappeared three times in the other patient. Recurrence rates for IP and FP did not differ significantly. All primary and recurrent FP analyzed in this study were HPV‐positive (4 of 4 initial FP, 2 of 2 recurrent FP). HPV 6 and 11 were detected in recurrent FP (each in 1 of 2 FP, 50%). Additionally, both of the recurrent FP presented low‐ to moderate‐grade dysplasia on the basis of histological examinations. Two of the seven patients presented with co‐infection of two different HPV genotypes at the time of initial diagnosis. Both patients did not develop tumor recurrences. Therefore, as described for IP patients, co‐infections with multiple HPV types did not increase the risk for recurrence of FP in this cohort of patients (*p* = 0.4667).

**FIGURE 2:**
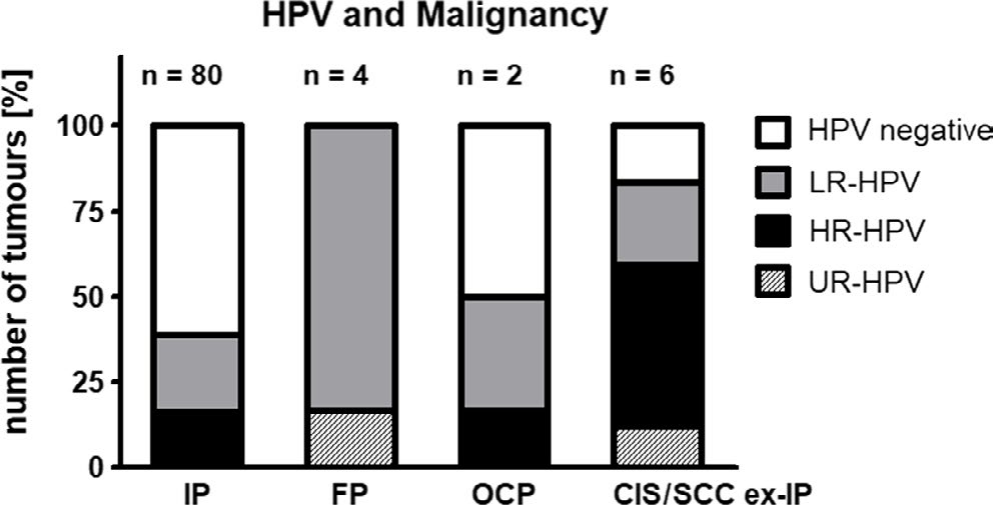
Carcinoma ex‐papilloma patients display a high rate of high‐risk human papilloma virus (HPV) infection. This graph shows the number of inverted (IP), fungiform (FP), oncocytic papilloma (OCP), and carcinoma in situ (CIS) / squamous cell carcinoma (SCC) ex‐IP that were either HPV‐negative or positive for low‐risk (LR), high‐risk (HR), or undetermined‐risk (UR) HPV. HPV rates were weighted to account for co‐infections with multiple HPV types. IP and OCP display a very similar pattern concerning HPV rates: 61.3% to 50% of the papillomas were HPV‐negative, 22.5% to 33.3% positive for LR‐HPV and 15.5% to 16.7% positive for HR‐HPV. In contrast, all investigated FP were HPV‐positive, yet none displayed HR‐HPV. In this context, it is noteworthy, that all malignant tumors arose from IP, which also displayed HR‐HPV infection (in contrast to FP, which displayed only LR‐/UR‐HPV infections). However, one also has to note that the patient numbers for FP, OCP, and malignomas are not very high. Additionally, the figure illustrates the high rate of HR‐HPV in patients with CIS or SCC ex‐IP (47.6%) in contrast to patients with benign lesions (max. 16.7%). Taken together, these data may support the hypothesis that especially HR‐HPV infection promotes malignant progression

As mentioned, two patients presented with oncocytic papillomas (OCP). One was HPV‐negative, the other one was HPV‐positive (1 of 2 OCP, 50%) (Figure 2). The HPV‐positive OCP displayed co‐infection with HPV 6, 16, and 42.^2^ Neither patient suffered tumor recurrence.

### 3.3 Carcinoma ex‐papilloma patients displayed a high rate of HR‐HPV over the course of their disease. HPV DNA was detected foremost in papillomas prior to malignant transformation or in the benign areas of SCC ex‐papilloma.

Six patients presented with malignant tumors ex‐papilloma: one patient with carcinoma in situ (CIS), five patients with squamous cell carcinoma (SCC). All of these malignant tumors developed from IP (none from FP or OCP). Two women and four men were affected. Except for one patient, all of them presented with advanced stage tumors, affecting at least three paranasal sinuses. Two patients (33.3%) presented with non‐resectable tumors. Five of the six patients with malignant tumors ex‐papilloma were initially diagnosed in our hospital. Therefore, the tissue samples of the primary tumors could be analyzed for HPV DNA. Two of them were positive for high‐risk HPV 16 (40%) at the time of initial diagnosis; three were HPV‐negative (60%) at that point.

When looking at the course of the disease of the six patients with malignant tumors (including initial diagnosis as well as recurrent/persisting malignant tumors), five of the six patients displayed HPV positivity during their oncologic course (83.3%). Four cases presented with high‐risk HPV infection (two patients with HPV 16, one patient with HPV 45, and one patient with HPV 66). The last HPV‐positive patient showed a co‐infection with HPV 90, for which the risk classification is still under discussion, and HPV 6 (Figure 2).

There were four patients with malignant tumors, for whom different regions of the same tumor could be investigated for HPV infection. That means, that tissue was harvested separately from the benign part of the tumor (sometimes displaying dysplasia but not yet carcinoma) and from the malignant part. These benign and malignant parts of the same tumor were then compared with regard to HPV infection. Three of the four cases were HPV‐positive in the benign part of the tumor, but no virus could be detected in the SCC part. For one patient, it was the other way round.

All of the six patients displayed malignancy at the time of initial diagnosis but for one exception: one patient presented with benign papilloma initially and returned after years with recurrent SCC ex‐papilloma. In this patient, HPV 16 DNA was detected in the primary tumor (IP) but not later in the SCC.

In regard to the other risk factors, two patients with malignant tumors ex‐IP were active smokers (two of six patients, 30%). In comparison to our earlier study, 33.3% of male benign IP patients and 38.5% of female IP patients were active smokers or had smoked before.^2^ Additionally, one patient with malignoma reported regular alcohol consumption, another patient high blood pressure, and a third patient an inhalation allergy.

## 4 DISCUSSION

Schneiderian papillomas are rare and usually benign tumors that arise from the sinonasal Schneiderian membrane. In the retrospective study presented in this article, we focused on the possible impact of HPV infection on tumor recurrence and on malignant progression of sinonasal papillomas. A cohort of 107 patients with 166 tumors was investigated. In decreasing number of frequency, patients were diagnosed with inverted papilloma (IP) (91 patients = 85%), fungiform papilloma (FP) (7 patients = 6.5%), and oncocytic papilloma (OCP) (2 patients = 1.9%). For one patient, papilloma entity (i.e., inverted vs. fungiform) could not be clearly determined. Malignant progression is a rare but severe complication of Schneiderian papillomas; therefore, risk factors and predictors for carcinogenesis have been subject to controversial discussions. The study presented here includes six patients with malignant tumors ex‐papilloma, that is, one patient with carcinoma in situ (CIS) and five patients with squamous cell carcinoma (SCC). Tissue samples from 12 malignant tumors could be gathered from these six patients. All malignant tumors arose from IP (none from FP or OCP). Gender did not seem to have an important impact on malignant progression of Schneiderian papillomas^20^: the male to female ratio of malignant tumors in our patient cohort was 2: 1 (3.5: 1 for benign IP, *p* = 0.6161). Additionally, we could not confirm some of the previously described possible risk factors, such as higher age and smoking^2^, ^20^, ^21^, ^22^, ^23^: patients with malignant lesions displayed a median age of 55 years at initial diagnosis (59 years for patients with benign IP); 30% of carcinoma ex‐papilloma patients were smokers (compared to 33.3% of male benign IP patients and 38.5% of female IP patients).

Our previous analysis found that two patients showed dysplasia in FP (not yet CIS). Both suffered from recurrent tumors.^2^ In the study presented here, almost every SCC ex‐IP also displayed areas with low‐ to high‐grade dysplasia. Therefore, dysplasia should alert the clinician—to look out for tumor recurrences—and the pathologist—to thoroughly evaluate of the whole papilloma (not to miss SCC parts within the tumor).

Human papillomaviruses (HPV) are DNA viruses that infect differentiating skin and mucous membrane cells. Through expression of viral oncogenes they can induce uncontrolled cell proliferation and even malignant progression.^4^, ^5^ The main mode of infection is probably through direct skin and mucosal contact. HPV types are classified according to their cancerogenic potential from low‐risk (LR‐HPV) to high‐risk types (HR‐HPV) (Table 1).^6^, ^7^, ^8^

The role of HPV infection as a risk or prognostic factor for recurrence rates or rates of malignant progression of sinonasal papillomas is still under controversial discussion. Some authors have observed that recurrence occurs more often in patients infected with HPV,^13^, ^14^, ^16^ while others were not as convinced and could not identify HPV as a significant predictor for recurrent papilloma.^17^, ^19^, ^24^ In our study, recurrent tumors were more often HPV‐positive than non‐recurrent IP (*p* = 0.0504): at initial diagnosis, 38.8% of inverted papillomas were HPV‐positive (31 of 80 IP), first recurrent tumors were 65% HPV‐positive (13 of 21 IP), and second recurrent tumors were 60% HPV‐positive (3 of 5 IP). Especially the rate of HPV 11, 16, 42, and 58 increased in recurrent papillomas, although none of these types individually showed significantly higher detection rates in recurrent IP (*p* = 0.3079 up to 0.8128) (Figure 1). However, when looking at the sum of low‐risk HP virus infections (especially HPV 6), they increased the risk for recurrent IP compared to the group of patients without HPV infection (*p* = 0.0385 for LR‐HPV and *p* = 0.0556 for HPV 6).

Several authors correlated HPV infection with malignant progression of Schneiderian papillomas.^14^, ^15^, ^16^, ^25^, ^26^, ^27^ Supporting this hypothesis, patients investigated in our study displayed higher HPV rates in CIS / SCC ex‐IP than in benign IP (83.3% and 38.8%, respectively, *p* = 0.0780). Additionally, patients with malignant tumors ex‐IP displayed higher rates of HR‐HPV than patients with benign inverted papillomas (66.7% vs. 23.8%, *p* = 0.0415). Detected HR‐HPV genotypes comprised HPV 16 (in two of six patients, 33.3%), HPV 45 and 66 (each in one patient, each 16.7%). Concerning malignant potential, both IP and OCP (but not FP) have been associated with malignant conditions, especially squamous cell carcinoma.^3^ In this context, we observed that patients with IP and OCP displayed higher rates of high‐risk HPV infection (15.5% and 16.7%, respectively) than patients with FP (fungiform papillomas neither progressed to carcinoma nor were they HR‐HPV positive) (*p* = 0.3333) (Figure 2). These data support the hypothesis that HPV infection, especially with HR‐HPV genotypes, promotes malignant transformation of sinonasal papillomas.^28^

Most data concerning the carcinogenic effect of high‐risk HPV was probably gathered in cervical cancer and its primary stages. Klug et al. present data from women undergoing routine PAP smears from December 1998 through 2000 in Germany (the timeframe and region of this study is comparable to our patient collective). ^29^ Similar to our data gathered from sinonasal papillomas, they found HPV 16 to be the most common genotype in regard to malignant progression of mucosal lesions of the cervix uteri. HPV 16 was found in roughly 60% of CIN 2 (cervical intraepithelial neoplasia) or worse lesions, followed by HPV 45 and 58 (almost 10%), by HPV 18, 31, 33, and 52 (roughly 7%), and by other less frequent HPV genotypes. These quotas mirror our data gathered from sinonasal papillomas very well. The fact sheet on human papillomas and related cancers, published by the Information Centre on HPV and Cancer, also underlines the predominance of HPV 16 over other HPV genotypes in low‐ through high‐grade dysplasia as well as cervical cancer in German women.^30^ However, their data show that HPV 18 is the second most common HPV genotype associated with cervical cancer. In contrast, data gathered from inverted papillomas and associated carcinomas show only few cases with HPV 18.^12^ However, Zhao et al. calculated a significant effect of HPV 18 infection on malignant progression of sinonasal papillomas in their meta‐analysis.^31^

In the head and neck area, many reports have been published on the probable role of HPV in oropharyngeal cancer: Ndiaye et al. published an extensive review with evidence that roughly 42% of oropharyngeal cancers are related to HPV in central Europe.^32^ As in cervical cancers, particularly HPV 16 seems to be important for carcinogenesis of head and neck squamous cell carcinomas.^33^

Returning to the data gathered in sinonasal papillomas in our study: there were four carcinoma ex‐IP patients, for whom different regions of the same tumor could be investigated for HPV infection: when comparing tissue derived from the benign part of the tumor (sometimes displaying dysplasia but not yet carcinoma) to parts of IP with proven malignancy, three of four patients were HPV‐positive in the benign part of the papilloma, but no virus could be detected in the SCC part of the same tumor. For one patient, it was the other way round. Furthermore, one patient initially presented with benign papilloma and returned after years with recurrent SCC ex‐papilloma. In this patient, HPV 16 DNA was detected in the primary tumor but not later in the SCC. Lawson et al. hypothesize that HPV can initiate development of papillomas but is not important for them to persist.^12^ The basic thought is, that HPV infects the sinonasal mucosa and induces genetic changes of the host's cells that lead to cell proliferation and formation of sinonasal papillomas. These tumors will continue growing even if the HPV infection is cleared by the immune system. This theory is commonly named the “hit and run” theory. A similar hit and run theory could be postulated for malignant tumors ex‐papillomas: if inverted papillomas are superinfected by HR‐HPV, this may lead to cellular changes that promote malignant progression even after the viral infection will be cleared.

To provide a wider picture, in regard to oncogenic HPV infections, two opposite modes of carcinogenic potential have long been discussed. On the one hand, there are authors providing evidence for a hit and run mode of HPV‐associated carcinogenesis.^34^ On the other hand, there are also data supporting the hypothesis that only persistent HPV infection has oncogenic effects.^35^ With increasing knowledge about epigenetic changes in recent years, a new possibility of viral carcinogenesis arose: some authors postulate virally induced epigenetic changes, that will remain, even if the original HPV infection is cleared by the immune system. In this scenario, viral oncogenesis will even be possible, if no HPV nucleic acids can be detected in patients’ samples.^36^ Indeed, epigenetic alterations such as DNA methylation and histone modification have already been found in HPV‐driven cervical carcinogenesis.^37^ In regard to Schneiderian papillomas, some authors have already shown that epigenetic changes promote malignant progression of sinonasal papillomas^38^; however, the extent of HPV‐association of these epigenetic alterations remains to be seen.

In conclusion, our data suggest that HPV infection, particularly infection with LR‐HPV, such as HPV 6, can be regarded as a risk factor for tumor recurrences of sinonasal papillomas. Furthermore, malignant progression of inverted papillomas seems to be a multi‐step process that may be supported (but not exclusively caused) by high‐risk HPV genotypes. Further studies are required to elucidate the potential impact of HPV on the disease course of inverted papillomas, which could help to improve therapeutic strategy and post‐therapeutic management for the patients.

## Data Availability

Data available on request due to privacy/ethical restrictions: The data that support the findings of this study are available on request from the first author [AP, email: anja.paehlervorderholte@krh.eu]. The data are not publicly available due to restrictions, for example, their containing information that could compromise the privacy of research participants. The data are also not available on a hospital server due to IT security reasons.

## References

[1] Vorasubin N , Vira D , Suh JD , Bhuta S , Wang MB . Schneiderian papillomas: comparative review of exophytic, oncocytic, and inverted papillomas. Am J Rhinol Allergy. 2013;4:287‐292. 10.2500/ajra.2013.27.3904 PMC3901443 23883810

[2] Paehler vor der Holte A , Fangk I , Glombitza S , Wilkens L , Welkoborsky HJ . Prognostic factors and risk factors for development and recurrence of sinonasal papillomas: potential role of different HPV subtypes. Eur Arch Otorhinolaryngol. 2020;3:767‐775. 10.1007/s00405-019-05747-4 31832748

[3] Barnes L . Schneiderian papillomas and nonsalivary glandular neoplasms of the head and neck. Mod Pathol. 2002;3:279‐297. 10.1186/s13027-015-0019-8 11904343

[4] D'Abramo CM , Archambault J . Small molecule inhibitors of human papillomavirus protein ‐ protein interactions. Open Virol J. 2011;80‐95. 21769307 10.2174/1874357901105010080 PMC3137155

[5] Klaes R , Woerner SM , Ridder R , et al. Detection of high‐risk cervical intraepithelial neoplasia and cervical cancer by amplification of transcripts derived from integrated papillomavirus oncogenes. Cancer Res. 1999;24:6132‐6136. 10626803

[6] Munoz N , Bosch FX , de Sanjos S , et al. Epidemiologic classification of human papillomavirus types associated with cervical cancer. N Engl J Med. 2003;6:518‐527. 10.1056/NEJMoa021641 12571259

[7] Varnai AD , Bollmann M , Bankfalvi A , et al. The spectrum of cervical diseases induced by low‐risk and undefined‐risk HPVS: Implications for Patient Management. Anticancer Res. 2007;1B:563‐570. 17348442

[8] Bouvard V , Baan R , Straif K , et al. A review of human carcinogens ‐ Part B: biological agents. Lancet Oncol. 2009;4:321‐322. 10.1016/s1470-2045(09)70096-8 19350698

[9] Fortes HR , von Ranke FM , Escuissato DL , et al. Recurrent respiratory papillomatosis: a state‐of‐the‐art review. Respir Med. 2017;126:116‐121. 28427542 10.1016/j.rmed.2017.03.030

[10] Loo SKF , Tang WYM . Warts (non‐genital). BMJ Clin Evidence. 2014;12. PMC4054795 24921240

[11] Strati K , Lambert PF . Human papillomavirus association with head and neck cancer: understanding virus biology and using it in the development of cancer diagnostics. Expert Opin Med Diagn. 2008;2:11‐20. 20419065 10.1517/17530059.2.1.11 PMC2858343

[12] Lawson W , Schlecht NF , Brandwein‐Gensler M . The role of the human papillomavirus in the pathogenesis of Schneiderian inverted papillomas: an analytic overview of the evidence. Head Neck Pathol. 2008;2:49‐59. 20614323 10.1007/s12105-008-0048-3 PMC2807546

[13] Ogura H , Fukushima K , Watanabe S . A high prevalence of human papillomavirus DNA in recurrent nasal papillomas. J Med Microbiol. 1996;3:162‐166. 10.1099/00222615-45-3-162 8810941

[14] Hwang CS , Yang HS , Hong MK . Detection of human papillomavirus (HPV) in sinonasal inverted papillomas using polymerase chain reaction (PCR). Am J Rhinol. 1998;5:363‐366. 10.2500/105065898780182499 9805538

[15] Scheel A , Lin GC , McHugh JB , et al. Human papillomavirus infection and biomarkers in sinonasal inverted papillomas: clinical significance and molecular mechanisms. Int Forum Allergy Rhinol. 2015;8:701‐707. 10.1002/alr.21524 PMC4526407 26077310

[16] Lin H , Lin D , Xiong XS . Roles of human papillomavirus infection and stathmin in the pathogenesis of sinonasal inverted papilloma. Head Neck. 2016;2:220‐224. 10.1002/hed.23864 25224680

[17] Kraft M , Simmen D , Casas R , Pfaltz M . Significance of human papillomavirus in sinonasal papillomas. J Laryngol Otol. 2001;9:709‐714. 10.1258/0022215011908955 11564296

[18] Justice JM , Davis KM , Saenz DA , Lanza DC . Evidence that human papillomavirus causes inverted papilloma is sparse. Int Forum Allergy Rhinol. 2014;12:995‐1001. 10.1002/alr.21358 25331985

[19] Jenko K , Kocjan B , Zidar N , et al. In inverted papillomas HPV more likely represents incidental colonization than an etiological factor. Virchows Arch. 2011;5:529‐538. 10.1007/s00428-011-1139-1 21912908

[20] Behringer S . Das invertierte Papillom der Nase und Nasennebenhöhlen. In: FreiDok plus Universitätsbibliothek Freiburg [database online]. Freiburg im Breisgau: Albert‐Ludwigs‐Universität; 2006 https://freidok.uni‐freiburg.de/fedora/objects/freidok:3028/datastreams/FILE1/content. Updated August, 2019. Accessed August 27, 2019

[21] Barnes L . Surgical pathology of the head and neck, 3rd edn New York, NY: CRC Press; 2008.

[22] Nachtigal D , Yoskovitch A , Frenkiel S , Braverman I , Rochon L . Unique characteristics of malignant Schneiderian papilloma. Otolaryngol Head Neck Surg. 1999;6:766‐771. 10.1053/hn.1999.v121.a98734 10580235

[23] Hong SL , Kim BH , Lee JH , Cho KS , Roh HJ . Smoking and malignancy in sinonasal inverted papilloma. Laryngoscope. 2013;5:1087‐1091. 10.1002/lary.23876 23619620

[24] Roh HJ , Mun SJ , Cho KS , Hong SL . Smoking, not human papilloma virus infection, is a risk factor for recurrence of sinonasal inverted papilloma. Am J Rhinol Allergy. 2016;2:79‐82. 10.2500/ajra.2016.30.4272 26980388

[25] Kashima HK , Kessis T , Hruban RH , Wu TC , Zinreich SJ , Shah KV . Human papillomavirus in sinonasal papillomas and squamous cell carcinoma. Laryngoscope. 1992;9:973‐976. 10.1288/00005537-199209000-00003 1325585

[26] McKay SP , Gregoire L , Lonardo F , Reidy P , Mathog RH , Lancaster WD . Human papillomavirus (HPV) transcripts in malignant inverted papilloma are from integrated HPV DNA. Laryngoscope. 2005;8:1428‐1431. 10.1097/01.mlg.0000168091.50584.b4 16094117

[27] Yamashita Y , Hasegawa M , Deng Z , et al. Human papillomavirus infection and immunohistochemical expression of cell cycle proteins pRb, p53, and p16INK4a in sinonasal diseases. Infect Agent Cancer. 2015;23. 10.1186/s13027-015-0019-8 PMC4524447 26244053

[28] Syrjanen KJ . HPV infections in benign and malignant sinonasal lesions. J Clin Pathol. 2003;3:174‐181. 10.1136/jcp.56.3.174 PMC1769909 12610092

[29] Klug SJ , Hukelmann M , Hollwitz B , et al. Prevalence of human papillomavirus types in women screened by cytology in germany. J Med Virol. 2007;79:616‐625. 17385693 10.1002/jmv.20863

[30] Information Centre on HPV and Cancer, Germany . Human Papillomavirus and related cancers, fact sheet 2018; 2019:06.

[31] Zhao R‐W , Guo Z‐Q , Zhang R‐X . Human papillomavirus infection and the malignant transformation of sinonasal inverted papilloma: a meta‐analysis. J Clin Virol. 2016;79:36‐43. 27085508 10.1016/j.jcv.2016.04.001

[32] Ndiaye C , Mena M , Alemany L , et al. HPV DNA, E6/E7 mRNA, and p16INK4a detection in head and neck cancers: a systematic review and meta‐analysis. Lancet Oncol. 2014;15(12):1319‐1331. 25439690 10.1016/S1470-2045(14)70471-1

[33] WHO Press . IARC monograph on the evaluation of carcinogenic risks to humans. Biological Agents. 2012;100B:255‐313. PMC4781184 23189750

[34] Iwasaka T , Hayashi Y , Yokoyama M , Hara K , Matsuo N , Sugimori H . Hit and run oncogenesis by human papillomavirus type 18 DNA. Acta Obstet Gynecol Scand. 1992;71:219‐223. 1317646 10.3109/00016349209009922

[35] Crook T , Morgenstern JP , Crawford L , Banks L . Continued expression of HPV‐16 E7 protein is required for maintenance of the transformed phenotype of cells co‐transformed by HPV‐16 plus EJ‐ras. EMBO J. 1989;8:513‐519. 2542020 10.1002/j.1460-2075.1989.tb03405.x PMC400835

[36] Niller HH , Wolf H , Minarovits J . Viral hit and run‐oncogenesis: genetic and epigenetic scenarios. Cancer Lett. 2011;305:200‐217. 20813452 10.1016/j.canlet.2010.08.007

[37] Soto D , Song C , McLaughlin‐Drubin ME . Epigenetic alterations in human papillomavirus‐associated cancers. Viruses. 2017;9(9):248. 10.3390/v9090248 PMC5618014 28862667

[38] Sahnane N , Ottini G , Turri‐Zanoni M , et al. Comprehensive analysis of HPV infection, EGFR exon 20 mutations and LINE1 hypomethylation as risk factors for malignant transformation of sinonasal‐inverted papilloma to squamous cell carcinoma. Int J Cancer. 2019;144:1313‐1320. 30411788 10.1002/ijc.31971

